# Inequalities in negative maternal perception of oral health among Brazilian children: findings from the 2023 National Oral Health Survey (SB Brasil 2023)

**DOI:** 10.1590/1980-549720260013.supl.1

**Published:** 2026-07-10

**Authors:** Rafaela Zazyki, Nathalia Ribeiro Jorge da Silva-Garcia, Sarah Arangurem Karam, Francine dos Santos Costa

**Affiliations:** I Universidade Federal de Pelotas, Postgraduate Program in Epidemiology, Faculdade de Medicina – Pelotas (RS), Brazil.; II Universidade Federal de Pelotas, Postgraduate Program in Dentistry, Faculdade de Odontologia – Pelotas (RS), Brazil.; III Universidade Católica de Pelotas, Postgraduate Program in Health Throughout the Life Cycle – Pelotas (RS), Brazil.

**Keywords:** Health inequalities, Oral health, Mother-child relationship, Perception

## Abstract

**Objective:**

To analyze socioeconomic inequalities in negative maternal perception of children's oral health in Brazil using data from the 2023 National Oral Health Survey (SB Brasil 2023).

**Methods:**

This cross-sectional study included 6,970 five-year-old children. Our outcome was a negative maternal perception of child oral health. Inequalities were assessed by sex, race/skin color, maternal education, region of the country and wealth index. Complex measures of inequality, Slope Index of Inequality (SII) and Concentration Index (CIX) were estimated using ordered stratification. For interpretation, the estimates were multiplied by 100.

**Results:**

The prevalence of a negative maternal perception of child oral health was 30.4% (95% confidence interval — 95%CI 27.1–33.9). It was higher among children whose mothers had ≤8 years of schooling (35.6%), were in the poorest wealth quintile (42.5%), and had caries experience (51.8%). The SII and CIX indicated marked inequalities for wealth quintiles (SII: −28.0; 95%CI −39.2; −16.7/ CIX: −10.3; 95%CI −17.4; −3.1) and maternal education (SII: −22.1; 95%CI −33.0; −11.2/ CIX: −7.9; 95%CI −14.5; −1.4). Higher prevalences were found in the North and Northeast regions and among Black and Brown children.

**Conclusion:**

Substantial socioeconomic inequalities persist in maternal perception of children's oral health in Brazil. The findings highlight the need for equity-oriented oral health policies and actions to address social and racial disparities.

## INTRODUCTION

Oral health is a fundamental component of overall health and well-being, influencing children's growth, development, and psychosocial dimensions of quality of life^1^. Despite advances in oral health promotion and the expansion of dental care, inequalities in the distribution of oral diseases remain a major public health concern, even in young children^2^. National epidemiological surveys from previous editions of the National Oral Health Survey (SB Brasil 2003 and 2010) have consistently demonstrated pronounced socioeconomic gradients in the polarization of untreated dental caries in early childhood, disproportionately affecting socioeconomically disadvantaged and racially marginalized groups^3,4^. These inequalities reflect the influence of broader social determinants of health, including income, maternal education, and access to services^5^.

Beyond clinical conditions, subjective indicators such as self-perceived oral health provide essential insights into how individuals experience and evaluate their oral status. Self-perception is shaped by both clinical needs and psychosocial and cultural contexts, and it is strongly associated with quality of life and use of dental services^6^. Importantly, poorer self-perceived oral health has been consistently reported among socially disadvantaged groups, reinforcing the role of subjective measures in revealing inequities that extend beyond clinical diagnoses^7,8^. Recent studies have highlighted the relevance of combining objective and subjective outcomes, such as parents' perception of the child's oral health, in oral health research. For instance, Karam et al.^6^ demonstrated that socioeconomic inequalities influence maternal perceptions of children's oral health independently of clinical status, underscoring the importance of subjective indicators in monitoring equity in oral health. However, evidence on maternal perception of children's oral health in the Brazilian population remains limited, particularly in nationally representative samples, after more than a decade without comprehensive surveys.

The 2023 edition of the SB Brasil survey provides an unprecedented opportunity to update national estimates and examine contemporary inequalities^8^ in both objective and subjective oral health outcomes. While clinical indicators have been widely reported in previous surveys, less attention has been devoted to the distribution of maternal perceptions of children's oral health across social groups. This information was not reported in SB Brasil 2010 for children, and a gap in the literature on this topic remains. Given this context, investigating socioeconomic inequalities in maternal perception of children's oral health using contemporary, nationally representative data is crucial to understanding how broader socioeconomic determinants continue to shape health experiences in Brazil. By using data from the 2023 SB Brasil survey, this study aimed to analyze the distribution of negative maternal perception of children's oral health and to examine how these perceptions are socially determined. This approach not only complements traditional clinical assessments, but also provides critical evidence to guide equity-oriented oral health policies and interventions.

## METHODS

The study was reported in accordance with the Strengthening the Reporting of Observational Studies in Epidemiology (STROBE) checklist^9^.

### Study design and population

We used data from the 2023 National Oral Health Survey (SB Brazil). This survey is a cross-sectional study collected in 2023 and 2024, the sample is nationally representative, and methodological details of the survey are available in the Technical Report^10^. For this analysis, we included children aged five with available information for the outcome, totaling 6,970 participants. For this age group, a questionnaire was completed by the parents or guardians, and a clinical examination was performed on the children by calibrated examiners.

### Variables

Our main outcome is the negative maternal perception of child oral health assessed through the question "*In general, how would you assess the child's oral health (teeth and gums)?*", with the following response options: very good, good, fair, poor, very poor, and don't know/ did not answer. For analytical purposes, we dichotomized the response options into positive perception (very good and good) and negative perception (fair, poor, and very poor). A total of 228 children were excluded from the analysis because the question was answered as "don't know" or was not answered.

The covariates of interest, representing equity dimensions, were child sex (female and male), child race/skin color, maternal education, regions of the country (North, Northeast, Central-West, Southeast and South), and the wealth index in quintiles. Information on race/skin color was reported by parents or guardians based on the child's skin color/race with the answer options White, Black, Asian, Brown, Indigenous. Maternal education was collected in years of study and categorized as zero at eight years, nine to 11 years, and 12 or more years of study. Principal component analysis (PCA) was performed to generate a wealth index, based on the inclusion of the variables: ownership of a refrigerator, ownership of a television set, ownership of sound system equipment, ownership of a microwave, ownership of a mobile phone, ownership of a landline phone, ownership of a washing machine, ownership of a dishwasher, ownership of a computer, ownership of internet connection, received a cash transfer benefit (*Bolsa Familia*), received other social benefit, has a car, and type of drinking water supply (piper water into dwelling — at least one room, piped water to yard/ plot only and no piped water). The PCA score was divided into quintiles to construct the wealth index.

Clinical variables were assessed according to the codes and criteria established by the World Health Organization (WHO)^11^. The decayed, missing and filled teeth index (DMFT) was calculated to assess dental caries experience. A DMFT index of 0 represents no caries experience (caries-free children). Untreated caries was defined as the sum of decayed teeth and filled teeth with caries. Children with a score of 1 or higher were considered to have untreated dental caries. We dichotomized the variables of untreated caries and dental caries experience to focus on investigating the relationship between the presence of decayed teeth or the experience of disease and maternal perception of children's oral health, rather than severity.

### Ethical aspects

The SB Brazil 2023 was approved by the National Research Ethics Committee, report number 4.823.054. An informed consent form was completed by all parents or guardians, and an assent form was completed by the children.

### Statistical analysis

We conducted a descriptive analysis presenting absolute and relative frequencies, and 95% confidence intervals to describe sample characteristics according to the outcome. The subgroups' findings were not presented if the number of observations in the category was lower than 25. A complex measure of inequality, the *Slope Index of Inequality* (SII), was calculated considering maternal education and wealth quintiles. SII represents the beta parameter of a logistic regression with maternal perception of the child's oral health as the dependent variable, and wealth quintiles or maternal education as the independent variables. We used logistic regression with predicted marginal probabilities based on the ranked socioeconomic distribution and multiplied the beta values by 100, presenting the prevalence gap in percentage points among children in the extreme of wealth quintiles and maternal education distribution. An SII value of 0 represents complete equality, negative values showing that the outcome was higher among more disadvantaged groups and positive values showing the outcome was higher among the more advantaged groups. The *Concentration Index* (CIX) was also calculated considering maternal education and wealth quintiles, and identifies relative inequalities showing how concentrated the outcome is across the socioeconomic distribution. A CIX equal to zero also represents complete equality, and the estimates were multiplied by 100 for interpretation. Proportions were estimated accounting for the complex sampling design of SB Brasil 2023, incorporating sampling weights, strata, and primary sampling units (PSUs). The inequality measures (SII and CIX) were estimated incorporating sampling weights and accounting for clustering. All analyses were performed using Stata version 18.5, through the survey (svy) commands.

#### Data availability statement:

All the data supporting the results of this study were published in the article itself.

## RESULTS

The total sample for this study was 6,970 individuals. The prevalence of negative maternal perception of children's oral health in the sample was 30.4% (95% confidence interval — 95%CI 27.1–33.9). Of the total, 44.4% (95%CI 40.0–48.9) are White children, 44.3% (95%CI 40.6–48.1) have mothers with nine to 11 years of education, and 46.8% (95%CI 43.2–50.5) have dental caries experience. The decayed component was the most prevalent, with 41.1% (95%CI 37.6–44.7) of the children aged five having untreated dental caries (Table 1).

**Table 1:** Frequency of characteristics of the study sample and prevalence of negative maternal perception of children's oral health. *Brazilian National Survey of Oral Health* (SB Brasil) 2023.

Variable		Sample Size		Negative perception of children's oral health % (95%CI)
		n (%)	95%CI	
Child's sex (n=6,970)				
	Female	3,559 (48.8)	48.3–54.1	30.3 (26.0–34.9)
	Male	3,411 (51.2)	45.9–51.7	30.6 (27.0–34.5)
Race/ Skin color (n=6,882)				
	White	2,422 (44.4)	40.0–48.9	22.8 (18.3–28.1)
	Black	711 (10.5)	8.2–13.3	39.8 (30.6–49.9)
	Brown	3,655 (45.1)	41.2–49.1	34.9 (30.9–39.1)
	Yellow	71 (0.8)	0.5–1.4	52.0 (29.6–73.6)
	Indigenous	23 (0.4)	0.1–1.6	(–)
Maternal educational level in years (n=6,445)				
	0–8	2,234 (37.0)	32.5–41.7	35.6 (30.8–40.7)
	9–11	3,107 (44.3)	40.6–48.1	30.4 (26.1–35.0)
	12 or more	1,104 (18.7)	14.4–23.9	17.0 (12.1–23.3)
Wealth quintiles (n=6,137)				
	Poorest	1,266 (17.8)	14.7–21.4	42.5 (34.0–51.4)
	Second	1,436 (20.0)	16.8–23.7	33.0 (27.5–38.9)
	Third	1,415 (20.6)	17.7–23.7	31.8 (26.4–37.7)
	Fourth	1,061 (20.3)	17.7–23.1	30.2 (23.5–38.0)
	Wealthiest	959 (21.3)	15.8–28.2	14.1 (8.8–21.7)
Untreated dental caries (n=6,966)				
	No	3,853 (58.9)	55.3–62.4	12.5 (9.9–15.5)
	Yes	3,113 (41.1)	37.6–44.7	56.2 (52.1–60.2)
Dental caries experience (n=6,966)				
	No	3,475 (53.2)	49.5–56.8	11.6 (8.9–14.8)
	Yes	3,491 (46.8)	43.2–50.5	51.8 (47.9–55.8)
Regions of the country (n=6,970)				
	North	1,719 (10.9)	8.9–13.3	31.1 (26.7–35.8)
	Northeast	2,579 (28.1)	24.1–32.5	36.8 (32.6–41.3)
	Central-West	1,038 (8.4)	6.7–10.6	36.1 (28.8–44.1)
	Southeast	786 (38.0)	31.9–44.4	26.6 (20.0–34.5)
	South	848 (14.6)	12.1–17.5	24.3 (18.9–30.6)

Missing values for maternal education (n=525), wealth quintiles (n=833), untreated dental caries (n=4), and dental caries experience (n=4); -: less than 25 observations.

Negative maternal perception of the child's oral health was similar between male and female children; however, the outcome was higher among children whose mothers had 0–8 years of education (35.6%), children from the Northeast region of the country (36.8%), and those in the poorest quintile of wealth (42.5%). For the clinical variables, negative maternal perception of the child's oral health was higher among children who had dental caries experience (51.8%) and who had untreated dental caries (56.2%) (Table 1).

Complex measures of inequalities for wealth quintiles and maternal education showed a wide difference between children in more disadvantage situations (the poorest quintile and the lowest maternal education) compared to children in the wealthiest quintile and whose mothers have higher education. The SII was −28.0 (95%CI −39.2; −16.7; p<0.01), and CIX was −10.3 (95%CI −17.4; −3.1; p<0.01), considering wealth quintiles. For maternal education, the SII was −22.1 (95%CI −33.0; −11.2; p<0.01) and the CIX −7.9 (95%CI −14.5; −1.4; p<0.01) (Supplementary Material 1).

**Figure 1:**
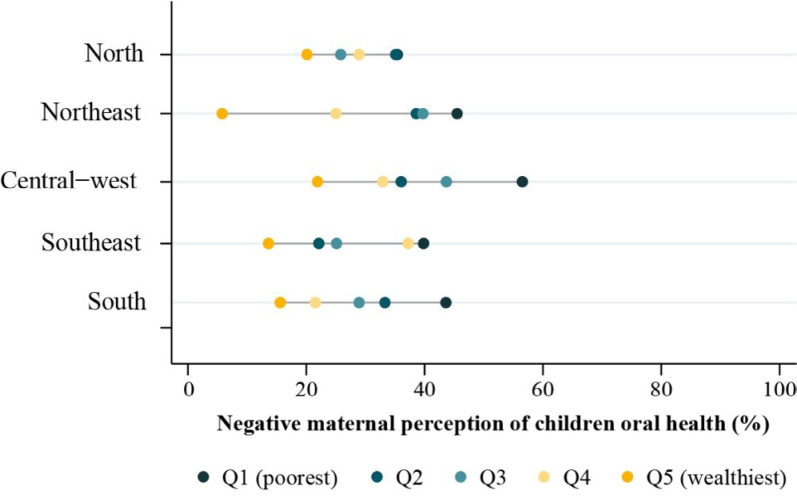
Regional Differences in Negative Maternal Perception of Children's Oral Health by wealth quintiles, SB Brasil, 2023.

As shown in Table 2, the prevalence of negative maternal perception of children's oral health varied across regions of the country. Overall, White children consistently presented lower prevalence compared to Black and Brown ones. Higher prevalence estimates were observed among Black children in the North, Northeast, and Central-West regions, whereas in the Southeast and South regions the highest estimates were observed among Brown children. For maternal education, although the lowest prevalences were observed among children of mothers with higher education across all regions of the country, the difference was significant only in the North and Northeast. The prevalence of the outcome was higher among children whose mothers have nine to 11 years of schooling in the North, Northeast, Central-West, and South regions, whereas, in the Southeast, it was higher among those whose mothers have zero to eight years of schooling (Table 2). In all regions of the country, the prevalence was higher in the poorest wealth quintile. The largest disparity appeared in the Northeast (Figure 1).

**Table 2:** Prevalence of negative maternal perception of children's oral health, according to Brazilian regions. *Brazilian National Survey of Oral Health* (SB Brasil) 2023.

Variable		North (n=1.719)	Northeast (n=2.579)	Central-West (n=1.038)	Southeast (n=786)	South (n=848)
		%(95%CI)	% (95%CI)	% (95%CI)	% (95%CI)	% (95%CI)
Child's sex						
	Female	29.4 (23.2–36.5)	36.8(30.7–43.3)	40.8 (30.7–51.8)	24.8 (17.1–34.4)	26.8 (20.4–34.3)
	Male	32.5 (25.4–40.4)	36.8 (32.3–41.6)	31.2 (24.5–38.7)	28.7 (20.9–38.0)	21.7 (15.4–29.7)
Race/ Skin color						
	White	32.8 (26.5–39.8)	33.1 (25.5–41.8)	27.8 (17.3–41.4)	17.3 (10.6–27.0)	22.9 (16.5–30.9)
	Black	35.8 (18.5–57.9)	52.2 (36.3–67.6)	45.5 (28.1–64.1)	30.4 (17.1–48.1)	21.9 (7.1–50.7)
	Brown	29.3 (24.4–34.7)	34.4 (29.4–39.6)	41.0 (33.3–49.2)	38.4 (28.1–49.9)	28.8 (17.9–42.8)
	Yellow	(-)	(-)	(-)	(-)	(-)
	Indigenous	(-)	(-)	(-)	(-)	(-)
Maternal educational (years)						
	0–8	28.6 (19.5–39.9)	46.1 (38.6–53.8)	36.9 (26.8–48.2)	34.6 (25.1–45.5)	24.4 (15.9–35.5)
	9–11	34.3 (28.6–40.5)	33.7 (27.2–40.9)	41.4 (32.5–50.9)	23.3 (14.9–34.7)	29.0 (20.3–39.6)
	12 or more	17.2 (11.6–24.9)	19.4 (12.6–28.7)	24.9 (17.8–33.9)	15.7 (8.3–27.7)	12.9 (5.3–27.1)
Wealth quintiles						
	Poorest	35.4 (24.1–48.6)	45.5 (37.5–53.7)	56.5 (42.4–69.6)	39.8 (16.2–69.4)	43.6 (14.4–78.0)
	Second	35.1 (29.0–41.8)	38.6 (30.7–47.1)	36.0 (23.6–50.7)	22.1 (11.2–39.0)	33.3 (14.0–60.6)
	Third	25.8 (19.6–33.1)	39.7 (29.5–50.9)	43.7 (32.8–55.2)	25.1 (15.8–37.5)	28.9 (18.2–42.6)
	Fourth	28.9 (18.4–42.4)	25.0 (15.8–37.1)	32.9 (21.0–47.5)	37.2 (22.9–54.2)	21.5 (11.8–35.8)
	Wealthiest	20.1 (9.8–36.8)	5.8 (2.4–13.2)	21.9 (14.0–32.7)	13.6 (6.2–27.3)	15.6 (8.4–27.3)
Untreated dental caries						
	No	9.8 (5.8–16.3)	17.3 (13.0–22.5)	11.2 (6.0–19.8)	12.2 (7.9–18.3)	7.5 (3.9–13.7)
	Yes	46.4 (38.8–54.2)	59.1 (53.1–64.8)	58.8 (51.3–65.8)	58.6 (48.4–68.2)	53.2 (42.4–63.7)
Dental caries experience						
	No	9.7 (5.5–16.6)	17.4 (12.9–23.0)	7.6 (4.6–12.2)	10.9 (6.7–17.4)	6.0 (2.9–12.2)
	Yes	43.9 (37.4–50.7)	55.6 (49.8–61.2)	53.1 (44.9–61.2)	53.6 (44.0–62.9)	47.1 (37.2–57.3)

95%CI: 95% confidence interval; -: less than 25 observations.

**Figure 2:**
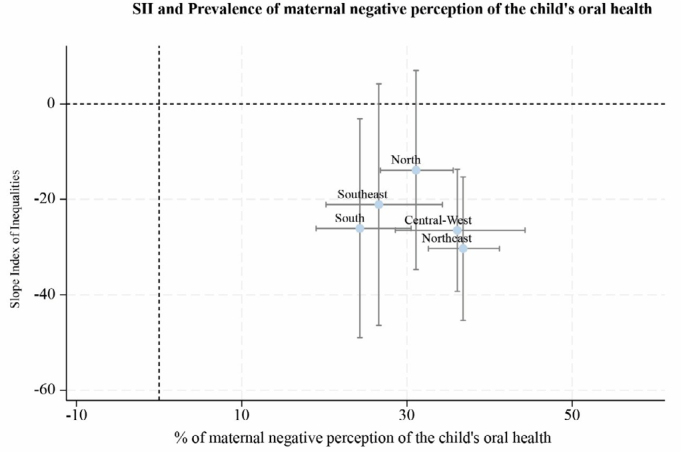
Regional differences in negative maternal perception of children's oral health, SB Brasil, 2023. The X-axis shows the prevalence (%) of negative maternal perception in each region, with 95% confidence intervals, and the Y-axis shows the Slope Index of Inequality (SII) by wealth quintiles, also with 95% confidence intervals. This presentation allows simultaneous visualization of both the overall prevalence and the magnitude of inequality across regions of the country.

**Figure 3:**
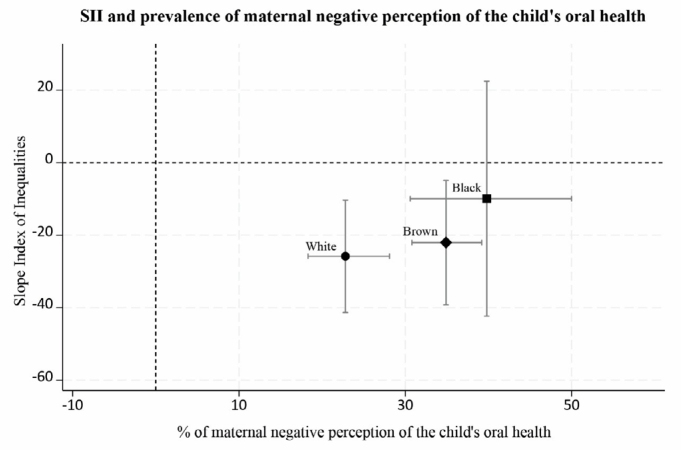
Inequalities in negative maternal perception of children's oral health according to children's racial/ ethnic group. The X-axis shows the prevalence (%) of negative maternal perception in each racial/ethnic group, with 95% confidence intervals, and the Y-axis shows the Slope Index of Inequality (SII) using wealth quintiles, also with 95% confidence intervals. This presentation allows simultaneous visualization of the overall prevalence and the magnitude of inequality between racial/skin color groups.

The relationship between the SII and the prevalence of negative perception of the child's oral health within each race/skin color subgroup indicates that inequality was greater among white children, although this group presented the lowest prevalence of the outcome. In contrast, Black children showed the highest prevalence, but with a smaller magnitude of inequality across wealth quintiles (Figures 2 and 3).

## DISCUSSION

The findings reveal persistent and marked socioeconomic disparities in maternal perception of children's oral health in the country. Negative perception was more frequent among socially disadvantaged groups. Additionally, the high prevalence of negative maternal perception among Black children, on average across wealth quintiles, represents a notable finding. These patterns mirror historical inequities in oral health conditions and reinforce the strong influence of socioeconomic determinants on how mothers perceive and experience their children's oral health. These groups, as reported in the literature, also experience higher rates of dental caries and untreated caries compared to children of mothers with higher education levels^12^, higher wealth quintiles^13,14^ and other race/skin color^15^.

It is important to acknowledge the limitations and strengths of this study to ensure accurate interpretation of the findings. First, the small number of observations of Indigenous and Asian children hindered robust analyses by race/skin color. Second, the outcome variable was based on maternal reports rather than children's self-perceptions, which may introduce information bias, as it can reflect maternal experiences and social views more than the children's own experiences^16^. This method of measurement may have led to underestimation or overestimation of the outcome, depending on the degree of agreement between maternal perception and the child's experience. However, given the age range of the children evaluated, parental reports are a methodologically appropriate approach and are widely used in the literature^6,7,17^. Third, some regions of the country presented wide confidence intervals, which reduces the precision of the estimates.

Another important limitation is the absence of information on maternal race/skin color, which may have restricted a more comprehensive understanding of the observed inequalities. A mother's racial/ethnic background influences her social experiences, interactions with institutions, and access to healthcare. These aspects can impact her caregiving methods, health-seeking behaviors, and perceptions of her child's health, ultimately affecting child health outcomes. Consequently, focusing only on the child's race/ skin color might cause us to overlook the larger social environment that fosters health inequities^18,19^.

The use of a subjective question can be a limitation because it does not allow definitive conclusions about the worst oral health conditions in children and is less precise than clinical variables. However, this approach can be used as a proxy for children's oral health when clinical exams are not possible; using a subjective question is more economical, practical and does not require clinical instruments^20,21^. Furthermore, in this sample, the prevalence of negative maternal perception of children's oral health was found to be four times higher among children with untreated dental caries compared to those without. On the other hand, the strengths include the use of nationally representative data, given that the study presents methodological robustness, a reliable sampling process, standardized and calibrated examiners, broad population coverage, and recent data.

Our study highlights a significant gap between the poorest and wealthiest income groups, as well as between the least and most educated groups, in negative maternal perception of children's oral health. This finding is consistent with the literature, which shows that individuals with the lowest income and education levels face greater barriers to accessing dental services when experiencing dental pain or caries^22^. Such barriers include financial constraints for traveling to health facilities, paying for treatment, or accessing care, and these populations also frequently have lower levels of health literacy^23^. Furthermore, adverse outcomes such as the presence of caries^15^, dental pain^24^, and lower frequency of dental visits^25^ are all associated with negative maternal perception of children's oral health.

Considering each region of the country, the highest prevalence of negative maternal perception of children's oral health was observed among Black and Browm race/skin color, but important regional differences in prevalence were identified between the North, Northeast, and Central-West regions and the South and Southeast regions. A similar pattern was observed across maternal education levels. The highest prevalence of negative maternal perception of children's oral health was observed in the Northeast, while the lowest was in the South. Similarly, the highest prevalence of untreated caries and dental caries experience was also in the Northeast. This region is characterized by the lowest income level of the country^26^ and high social and economic vulnerability, which likely drives worse oral health outcomes among children and worse maternal perception. The persistent social and economic heterogeneity across the country not only reinforces inequalities in access to health services^27^ but also suggests that the variation in the implementation of important oral health programs may be another contributing factor to the worst maternal perception in this region^28^.

The prevalence of negative maternal perceptions reveals a marked disparity among race/skin color in Brazil. Black children exhibited a higher prevalence than White ones, although the difference was smaller when compared to Brown children. This pervasive pattern of negative perception underscores the profound structural inequalities and racism experienced by this population, which negatively impacts numerous dimensions of their health and life outcomes^6,29^. In many cases, prejudice is also institutional, occurring within the health system itself, where those responsible for providing care may reproduce discriminatory practices and unequal treatment^30–32^. This further worsens access barriers and lowers the quality of care received by Black and Brown populations. Such a context underscores the importance of public policies specifically designed to promote equity in accessing oral health services, as well as the effective implementation of existing policies, such as the National Policy for the Comprehensive Health of the Black Population^33,34^.

In conclusion, this study provides nationally representative evidence of persistent socioeconomic and racial inequalities in maternal perception of children's oral health in Brazil. The findings demonstrate that maternal education, household wealth, and race/skin color remain key determinants of how mothers assess their children's oral health status, reflecting broader socioeconomic inequities that extend beyond clinical conditions. These results reinforce the importance of integrating equity-oriented approaches into oral health surveillance and policy planning, particularly in regions with greater socioeconomic vulnerability. Expanding access to preventive care, strengthening oral health literacy among caregivers, and ensuring culturally sensitive and inclusive practices within the Brazilian Unified Health System (SUS) are essential strategies to promote fairer and more equitable oral health outcomes for all Brazilian children.
