# Supplementary material for: Inequalities in negative maternal perception of oral health among Brazilian children: findings from the 2023 National Oral Health Survey (SB Brasil 2023)
Source: Rev Bras Epidemiol. 2026 Jul 10;29(Suppl 1):e260013supl1. doi: 10.1590/1980-549720260013.supl.1 (PMC13360983; doi:10.1590/1980-549720260013.supl.1)
Supplement: Supplementary Material 1 [file 1980-5497-rbepid-29-suppl1-e260013supl1-supp01.doc]

**Supplementary Table 1. Concentration Index (CIX) for negative maternal perception of children’s oral health by regions of the country and children’s race/skin color, using wealth quintiles as the socioeconomic ranking variable.**

|  | **Wealth quintiles** | | **Maternal education** | |
| --- | --- | --- | --- | --- |
| **Variables** | **Slope Index of inequalities (95% CI)** | **Concentration Index (95% CI)** | **Slope Index of inequalities (95% CI)** | **Concentration Index (95% CI)** |
| **Regions of the country** |  |  |  |  |
| North | -13.9(-34.7;7.0) | -9.8(-19.5;0.0) | -3.5(-27.5;20.5) | 2.2(-9.2;13.5) |
| Northeast | -30.3(-45.4; -15.3) | -8.8(-16.2; -1.4) | -31.1(-44.6; -17.6) | -8.2(-14.3; -2.1) |
| Central-West | -26.5(-39.3; -13.7) | -10.9(-17.4; -4.5) | -10.0(-26.5;6.6) | -1.4(-7.7;4.9) |
| Southeast | -21.1(-46.4;4.2) | -4.5(-22.4;13.4) | -27.1(-47.3; -6.9) | -7.9(-23.6;7.9) |
| South | -26.1(-49.0; -3.1) | -6.2(-22.9;10.5) | -6.9(-24.3;10.5) | -1.1(-12.5;10.4) |
| **Race/ Skin color** |  |  |  |  |
| White | -25.8(-41.3; -10.3) | -15.4(-26.2; -4.6) | -19.2(-33.3; -6.5) | -9.0(-20.3;2.3) |
| Black | -9.9(-42.3;22.5) | -3.3(-14.5;8.0) | -15.6(-41.4;10.2) | -7.4(-17.8;3.1) |
| Brown | -22.0(-39.2; -4.9) | -4.7(-13.7;4.4) | -18.9(-37.8;0.0) | -3.5(-11.6;4.5) |
| Total | -28.0(-39.2; -16.7) | -10.3(-17.4; -3.1) | -22.1(-33.0; -11.2) | -7.9(-14.5; -1.4) |

95%CI: 95% Confidence Interval.

Note: The Yellow and Indigenous groups were not presented in the table due to the small number of observations (fewer than 25 children in each group).
