# Supplementary material for: Inequalities in negative maternal perception of oral health among Brazilian children: findings from the 2023 National Oral Health Survey (SB Brasil 2023)
Source: Rev Bras Epidemiol. 2026 Jul 10;29(Suppl 1):e260013supl1. doi: 10.1590/1980-549720260013.supl.1 (PMC13360983; doi:10.1590/1980-549720260013.supl.1)
Supplement: Translation

# Translation: Desigualdades na percepção materna negativa sobre a saúde bucal entre crianças brasileiras: resultados da Pesquisa Nacional de Saúde Bucal 2023 (SB Brasil 2023)

**DOI:** 10.1590/1980-549720260013.supl.1.2

**Authors:** Rafaela Zazyki, Nathalia Ribeiro Jorge da Silva-Garcia, Sarah Arangurem Karam, Francine dos Santos Costa

**Affiliations:** I Universidade Federal de Pelotas, Programa de Pós-Graduação em Epidemiologia, Faculdade de Medicina – Pelotas (RS), Brasil.; II Universidade Federal de Pelotas, Programa de Pós-Graduação em Odontologia, Faculdade de Odontologia – Pelotas (RS), Brasil.; III Universidade Católica de Pelotas, Programa de Pós-Graduação em Saúde no Ciclo Vital – Pelotas (RS), Brasil.

**Keywords:** Desigualdades de saúde, Saúde bucal, Relações mãe-filho, Percepção

## Abstract

**Objetivo:**

Analisar as desigualdades socioeconômicas na percepção materna negativa sobre a saúde bucal das crianças no Brasil, utilizando dados da Pesquisa Nacional de Saúde Bucal 2023 (SB Brasil 2023).

**Métodos:**

Estudo transversal que incluiu 6.970 crianças de cinco anos de idade. O desfecho foi a percepção materna negativa sobre a saúde bucal infantil. As desigualdades foram avaliadas segundo sexo, raça/cor da pele, escolaridade materna, região do país e índice de riqueza. Foram estimadas medidas complexas de desigualdade, Índice de Inclinação de Desigualdade (*Slope Index of Inequality* – SII) e Índice de Concentração (CIX), utilizando estratificação ordenada. Para interpretação, as estimativas foram multiplicadas por 100.

**Resultados:**

A prevalência de percepção materna negativa da saúde bucal infantil foi de 30,4% (intervalo de confiança de 95% — IC95% 27,1–33,9). Foi mais elevada entre crianças cujas mães tinham ≤8 anos de escolaridade (35,6%), pertenciam ao quintil mais pobre de riqueza (42,5%) e apresentavam experiência de cárie dentária (51,8%). O SII e o CIX indicaram desigualdades marcantes segundo quintis de riqueza (SII: −28,0; IC95%: −39,2; −16,7/ CIX: −10,3; IC95%: −17,4; −3,1) e escolaridade materna (SII: −22,1; IC95%: −33,0; −11,2/ CIX: −7,9; IC95%: −14,5; −1,4). Maiores prevalências foram observadas nas regiões Norte e Nordeste e entre crianças pretas e pardas.

**Conclusão:**

Desigualdades socioeconômicas substanciais persistem na percepção materna da saúde bucal infantil no Brasil. Os achados reforçam a necessidade de políticas e ações de saúde bucal orientadas à equidade para enfrentar desigualdades sociais e raciais.

## INTRODUÇÃO

A saúde bucal constitui componente fundamental da saúde e do bem-estar geral, influenciando o crescimento, o desenvolvimento e as dimensões psicossociais da qualidade de vida das crianças^1^. Apesar dos avanços na promoção da saúde bucal e da ampliação do acesso ao atendimento odontológico, as desigualdades na distribuição das doenças bucais permanecem como importante problema de saúde pública, inclusive em crianças pequenas^2^. Levantamentos epidemiológicos nacionais, provenientes de edições anteriores da Pesquisa Nacional de Saúde Bucal (SB Brasil 2003 e 2010), têm demonstrado, de forma consistente, acentuados gradientes socioeconômicos na polarização da cárie dentária não tratada na primeira infância, com impacto desproporcional sobre grupos socioeconomicamente desfavorecidos e racialmente marginalizados^3,4^. Essas desigualdades refletem a influência de determinantes sociais de saúde mais amplos, incluindo renda, escolaridade materna e acesso aos serviços de saúde^5^.

Além das condições clínicas, indicadores subjetivos, como a autopercepção da saúde bucal, fornecem informações essenciais sobre a forma como os indivíduos vivenciam e avaliam seu estado bucal. A autopercepção é influenciada tanto por necessidades clínicas quanto por contextos psicossociais e culturais, estando fortemente associada à qualidade de vida e à utilização de serviços odontológicos^6^. Destaca-se que uma pior autopercepção da saúde bucal tem sido consistentemente relatada entre grupos socialmente desfavorecidos, reforçando o papel das medidas subjetivas na identificação de desigualdades que transcendem os diagnósticos clínicos^7,8^. Estudos recentes têm evidenciado a relevância da combinação de desfechos objetivos e subjetivos, como a percepção dos pais acerca da saúde bucal dos filhos, em pesquisas na área. Nesse contexto, Karam et al.^6^ demonstraram que as desigualdades socioeconômicas influenciam a percepção materna da saúde bucal infantil independentemente do quadro clínico, ressaltando a importância dos indicadores subjetivos no monitoramento da equidade em saúde bucal. Entretanto, as evidências sobre a percepção materna da saúde bucal infantil na população brasileira ainda são limitadas, especialmente em amostras representativas em nível nacional, após mais de uma década sem a realização de inquéritos abrangentes.

A edição de 2023 do SB Brasil oferece uma oportunidade inédita para a atualização das estimativas nacionais e para o exame das desigualdades contemporâneas^8^ em desfechos de saúde bucal, tanto objetivos quanto subjetivos. Embora os indicadores clínicos tenham sido amplamente relatados em investigações anteriores, menor atenção tem sido dedicada à distribuição da percepção materna sobre a saúde bucal infantil entre diferentes grupos sociais. Ressalta-se que essa informação não foi reportada no SB Brasil 2010 para crianças, persistindo, assim, uma lacuna na literatura sobre o tema. Nesse contexto, a investigação das desigualdades socioeconômicas na percepção materna sobre a saúde bucal infantil, com base em dados contemporâneos e representativos em nível nacional, mostra-se fundamental para a compreensão de como determinantes socioeconômicos mais amplos continuam a moldar as experiências de saúde no Brasil. Utilizando dados do SB Brasil 2023, o presente estudo teve como objetivo analisar a distribuição da percepção materna negativa sobre a saúde bucal infantil e examinar de que forma essas percepções são socialmente determinadas. Essa abordagem não apenas complementa as avaliações clínicas tradicionais, como também fornece evidências essenciais para subsidiar políticas e intervenções em saúde bucal com enfoque na equidade.

## MÉTODOS

O estudo foi reportado de acordo com a lista de verificação *Strengthening the Reporting of Observational Studies in Epidemiology* (STROBE)^9^.

### Desenho do estudo e população

Foram utilizados dados da Pesquisa Nacional de Saúde Bucal de 2023 (SB Brasil). Trata-se de um estudo transversal, conduzido entre 2023 e 2024, com amostra representativa em nível nacional. Os detalhes metodológicos da pesquisa encontram-se descritos no Relatório Técnico^10^. Para a presente análise, foram incluídas crianças de cinco anos de idade com informações disponíveis sobre o desfecho, totalizando 6.970 participantes. Para essa faixa etária, um questionário foi respondido pelos pais ou responsáveis, e o exame clínico odontológico foi realizado nas crianças por examinadores previamente calibrados.

### Variáveis

O desfecho principal foi a percepção materna negativa da saúde bucal infantil, avaliada por meio da pergunta: "Em geral, como você avaliaria a saúde bucal (dentes e gengivas) da criança?", com as seguintes opções de resposta: muito boa, boa, regular, ruim, muito ruim e não sei/não respondeu. Para fins analíticos, as respostas foram dicotomizadas em percepção positiva (muito boa e boa) e percepção negativa (regular, ruim e muito ruim).

Um total de 228 crianças foi excluído da análise devido à resposta "não sei" ou à ausência de resposta para essa variável.

As covariáveis de interesse, representativas das dimensões da equidade, incluíram sexo da criança (feminino e masculino), raça/cor da pele da criança, escolaridade materna, regiões do país (Norte, Nordeste, Centro-Oeste, Sudeste e Sul) e índice de riqueza em quintis. As informações sobre raça/cor da pele foram relatadas pelos pais ou responsáveis, com base na classificação da criança, considerando as seguintes categorias: branca, negra, asiática, parda e indígena. A escolaridade materna foi mensurada em anos de estudo e categorizada em três grupos: de zero a oito anos, de nove a 11 anos e 12 anos ou mais de estudo. Para a construção do índice de riqueza, foi realizada análise de componentes principais (ACP), com base na inclusão das seguintes variáveis: posse de geladeira, televisão, aparelho de som, micro-ondas, telefone celular, telefone fixo, máquina de lavar roupa, lava-louças, computador, acesso à internet, recebimento de benefício de transferência de renda (Bolsa Família), recebimento de outros benefícios sociais, posse de automóvel e tipo de abastecimento de água potável (água encanada na residência — em pelo menos um cômodo; água encanada apenas no quintal/terreno; e ausência de água encanada). A pontuação obtida pela ACP foi posteriormente categorizada em quintis para a construção do índice de riqueza.

As variáveis clínicas foram avaliadas de acordo com os códigos e critérios estabelecidos pela Organização Mundial da Saúde (OMS)^11^. O índice de dentes cariados, perdidos e obturados (CPOD) foi utilizado para avaliar a experiência de cárie dentária. Um índice CPOD igual a 0 indica ausência de cárie (crianças livres de cárie). A cárie não tratada foi definida como a soma de dentes cariados e dentes restaurados mas com presença de cárie. Crianças com valor igual ou superior a 1 foram classificadas como portadoras de cárie dentária não tratada. As variáveis de cárie não tratada e de experiência de cárie dentária foram dicotomizadas, com o objetivo de investigar a associação entre a presença de dentes cariados ou a experiência da doença e a percepção materna da saúde bucal das crianças, em detrimento da análise da gravidade.

### Aspectos éticos

O estudo SB Brasil 2023 foi aprovado pelo Comitê Nacional de Ética em Pesquisa, sob o parecer n° 4.823.054. O Termo de Consentimento Livre e Esclarecido foi obtido junto a todos os pais ou responsáveis, e o Termo de Assentimento foi obtido das crianças participantes.

### Análise estatística

Foi realizada análise descritiva, com apresentação de frequências absolutas e relativas, bem como intervalos de confiança de 95%, para caracterizar a amostra segundo o desfecho. Os resultados dos subgrupos não foram apresentados quando o número de observações na categoria foi inferior a 25. Uma medida complexa de desigualdade, o Índice Absoluto de Desigualdade (*Slope Index of Inequality* – SII), foi calculada considerando a escolaridade materna e os quintis de riqueza. O SII corresponde ao parâmetro beta de um modelo de regressão logística, tendo como variável dependente a percepção materna da saúde bucal da criança e, como variáveis independentes, os quintis de riqueza ou a escolaridade materna. Foram utilizadas probabilidades marginais previstas, com base na distribuição socioeconômica hierarquizada, e os coeficientes beta foram multiplicados por 100, sendo apresentados como diferença de prevalência, em pontos percentuais, entre crianças nos extremos da distribuição de quintis de riqueza e de escolaridade materna. Valor de SII igual a 0 indica igualdade completa; valores negativos indicam maior ocorrência do desfecho entre os grupos mais desfavorecidos, enquanto valores positivos indicam maior ocorrência entre os grupos mais favorecidos. O Índice de Concentração (*Concentration Index* – CIX) também foi estimado, considerando os quintis de escolaridade materna e de riqueza, permitindo a identificação de desigualdades relativas por meio do grau de concentração do desfecho ao longo da distribuição socioeconômica. Valor de CIX igual a 0 representa igualdade completa. As estimativas foram multiplicadas por 100 para fins de interpretação. As proporções foram estimadas considerando o desenho amostral complexo do SB Brasil 2023, com incorporação de pesos amostrais, estratos e unidades primárias de amostragem (UPAs). As medidas de desigualdade (SII e CIX) foram estimadas com incorporação dos pesos amostrais e consideração do efeito de agrupamento. Todas as análises foram realizadas no Stata, versão 18.5, por meio dos comandos *survey* (*svy*).

#### Declaração de disponibilidade de dados:

Todo o conjunto de dados que dá suporte aos resultados deste estudo foi publicado no próprio artigo.

## RESULTADOS

A amostra total do estudo foi composta por 6.970 indivíduos. A prevalência de percepção materna negativa da saúde bucal infantil foi de 30,4% (intervalo de confiança de 95% — IC95% 27,1–33,9). Do total, 44,4% (IC95% 40,0–48,9) eram crianças brancas, 44,3% (IC95% 40,6–48,1) tinham mães com nove a 11 anos de escolaridade e 46,8% (IC95% 43,2–50,5) apresentavam experiência de cárie dentária. O componente cariado foi o mais prevalente, com 41,1% (IC95% 37,6–44,7) das crianças de cinco anos apresentando cárie dentária não tratada (Tabela 1).

**Tabela 1:** Frequência das características da amostra do estudo e prevalência de percepção materna negativa da saúde bucal das crianças. Pesquisa Nacional de Saúde Bucal de 2023 (SB Brasil) 2023.

Variável		Tamanho da amostra		Percepção negativa da saúde bucal das crianças % (IC95%)
		n (%)	IC95%	
Sexo da criança (n=6.970)				
	Feminino	3.559 (48,8)	48,3–54,1	30,3 (26,0–34,9)
	Masculino	3.411 (51,2)	45,9–51,7	30,6 (27,0–34,5)
Raça/Cor da pele (n=6,882)				
	Branca	2.422 (44,4)	40,0–48,9	22,8 (18,3–28,1)
	Preta	711 (10,5)	8,2–13,3	39,8 (30,6–49,9)
	Parda	3.655 (45,1)	41,2–49,1	34,9 (30,9–39,1)
	Amarela	71 (0,8)	0,5–1,4	52,0 (29,6–73,6)
	Indígena	23 (0,4)	0,1–1,6	(–)
Escolaridade materna em anos (n=6.445)				
	0–8	2.234 (37,0)	32,5–41,7	35,6 (30,8–40,7)
	9–11	3.107 (44,3)	40,6–48,1	30,4 (26,1–35,0)
	12 ou mais	1.104 (18,7)	14,4–23,9	17,0 (12,1–23,3)
Quintis de riqueza (n=6.137)				
	Mais pobres	1.266 (17,8)	14,7–21,4	42,5 (34,0–51,4)
	Segundo	1.436 (20,0)	16,8–23,7	33,0 (27,5–38,9)
	Terceiro	1.415 (20,6)	17,7–23,7	31,8 (26,4–37,7)
	Quarto	1.061 (20,3)	17,7–23,1	30,2 (23,5–38,0)
	Mais ricos	959 (21,3)	15,8–28,2	14,1 (8,8–21,7)
Cárie dentária não tratada (n=6.966)				
	Não	3.853 (58,9)	55,3–62,4	12,5 (9,9–15,5)
	Sim	3.113 (41,1)	37,6–44,7	56,2 (52,1–60,2)
Experiência de cárie dentária (n=6.966)				
	Não	3.475 (53,2)	49,5–56,8	11,6 (8,9–14,8)
	Sim	3.491 (46,8)	43,2–50,5	51,8 (47,9–55,8)
Regiões do país (n=6.970)				
	Norte	1.719 (10,9)	8,9–13,3	31,1 (26,7–35,8)
	Nordeste	2.579 (28,1)	24,1–32,5	36,8 (32,6–41,3)
	Centro-Oeste	1.038 (8,4)	6,7–10,6	36,1 (28,8–44,1)
	Sudeste	786 (38,0)	31,9–44,4	26,6 (20,0–34,5)
	Sul	848 (14,6)	12,1–17,5	24,3 (18,9–30,6)

Valores ausentes para escolaridade materna (n=525), quintis de riqueza (n=833), cárie dentária não tratada (n=4) e experiência de cárie dentária (n=4); -: menos de 25 observações.

A percepção materna negativa da saúde bucal infantil foi semelhante entre meninos e meninas. No entanto, observou-se maior frequência desse desfecho entre crianças cujas mães apresentavam de 0 a oito anos de escolaridade (35,6%), residentes na região Nordeste do país (36,8%) e pertencentes ao quintil mais baixo de renda (42,5%). Em relação às variáveis clínicas, a percepção materna negativa da saúde bucal infantil foi mais elevada entre crianças com experiência de cárie dentária (51,8%) e entre aquelas com cárie dentária não tratada (56,2%) (Tabela 1).

As medidas complexas de desigualdade, considerando os quintis de riqueza e a escolaridade materna, evidenciaram diferenças expressivas entre crianças em maior situação de desvantagem (quintil mais pobre e menor escolaridade materna) em comparação àquelas pertencentes ao quintil mais rico e cujas mães apresentavam maior escolaridade. Para os quintis de riqueza, o SII foi de −28,0 (IC95% −39,2; −16,7; p<0,01) e o CIX de −10,3 (IC95% −17,4; −3,1; p<0,01). Em relação à escolaridade materna, o SII foi de −22,1 (IC95% −33,0; −11,2; p<0,01) e o CIX de −7,9 (IC95% −14,5; −1,4; p<0,01) (Material Suplementar 1).

Conforme apresentado na Tabela 2, a prevalência de percepção materna negativa da saúde bucal infantil variou entre as regiões do país. De modo geral, crianças brancas apresentaram prevalência consistentemente menor do que às crianças negras e pardas. Estimativas mais elevadas foram observadas entre crianças negras nas regiões Norte, Nordeste e Centro-Oeste, enquanto, nas regiões Sudeste e Sul, as maiores prevalências foram identificadas entre crianças pardas. Em relação à escolaridade materna, embora as menores prevalências tenham sido observadas entre filhos de mães com maior escolaridade em todas as regiões do país, essa diferença foi estatisticamente significativa apenas nas regiões Norte e Nordeste. A prevalência do desfecho foi maior entre crianças cujas mães apresentavam de nove a 11 anos de escolaridade nas regiões Norte, Nordeste, Centro-Oeste e Sul, enquanto, na região Sudeste, foi mais elevada entre aquelas cujas mães possuíam de zero a oito anos de estudo (Tabela 2). Em todas as regiões do país, observou-se maior prevalência no quintil de renda mais baixo, sendo a maior disparidade identificada na região Nordeste (Figura 1).

**Tabela 2:** Prevalência de percepção materna negativa da saúde bucal das crianças, segundo regiões do Brasil. Pesquisa Nacional de Saúde Bucal de 2023 (SB Brasil) 2023.

Variável		Norte (n=1.719)	Nordeste (n=2.579)	Centro-Oeste (n=1.038)	Sudeste (n=786)	Sul (n=848)
		%(IC 95%)	% (IC 95%)	% (IC 95%)	% (IC 95%)	% (IC 95%)
Sexo da criança						
	Feminino	29,4 (23,2–36,5)	36,8(30,7–43,3)	40,8 (30,7–51,8)	24,8 (17,1–34,4)	26,8 (20,4–34,3)
	Masculino	32,5 (25,4–40,4)	36,8 (32,3–41,6)	31,2 (24,5–38,7)	28,7 (20,9–38,0)	21,7 (15,4–29,7)
Raça/Cor da pele						
	Branca	32,8 (26,5–39,8)	33,1 (25,5–41,8)	27,8 (17,3–41,4)	17,3 (10,6–27,0)	22,9 (16,5–30,9)
	Preta	35,8 (18,5–57,9)	52,2 (36,3–67,6)	45,5 (28,1–64,1)	30,4 (17,1–48,1)	21,9 (7,1–50,7)
	Parda	29,3 (24,4–34,7)	34,4 (29,4–39,6)	41,0 (33,3–49,2)	38,4 (28,1–49,9)	28,8 (17,9–42,8)
	Amarela	(-)	(-)	(-)	(-)	(-)
	Indígena	(-)	(-)	(-)	(-)	(-)
Escolaridade materna (anos)						
	0–8	28,6 (19,5–39,9)	46,1 (38,6–53,8)	36,9 (26,8–48,2)	34,6 (25,1–45,5)	24,4 (15,9–35,5)
	9–11	34,3 (28,6–40,5)	33,7 (27,2–40,9)	41,4 (32,5–50,9)	23,3 (14,9–34,7)	29,0 (20,3–39,6)
	12 ou mais	17,2 (11,6–24,9)	19,4 (12,6–28,7)	24,9 (17,8–33,9)	15,7 (8,3–27,7)	12,9 (5,3–27,1)
Quintis de riqueza						
	Mais pobres	35,4 (24,1–48,6)	45,5 (37,5–53,7)	56,5 (42,4–69,6)	39,8 (16,2–69,4)	43,6 (14,4–78,0)
	Segundo	35,1 (29,0–41,8)	38,6 (30,7–47,1)	36,0 (23,6–50,7)	22,1 (11,2–39,0)	33,3 (14,0–60,6)
	Terceiro	25,8 (19,6–33,1)	39,7 (29,5–50,9)	43,7 (32,8–55,2)	25,1 (15,8–37,5)	28,9 (18,2–42,6)
	Quarto	28,9 (18,4–42,4)	25,0 (15,8–37,1)	32,9 (21,0–47,5)	37,2 (22,9–54,2)	21,5 (11,8–35,8)
	Mais ricos	20,1 (9,8–36,8)	5,8 (2,4–13,2)	21,9 (14,0–32,7)	13,6 (6,2–27,3)	15,6 (8,4–27,3)
Cárie dentária não tratada						
	Não	9,8 (5,8–16,3)	17,3 (13,0–22,5)	11,2 (6,0–19,8)	12,2 (7,9–18,3)	7,5 (3,9–13,7)
	Sim	46,4 (38,8–54,2)	59,1 (53,1–64,8)	58,8 (51,3–65,8)	58,6 (48,4–68,2)	53,2 (42,4–63,7)
Experiência de cárie dentária						
	Não	9,7 (5,5–16,6)	17,4 (12,9–23,0)	7,6 (4,6–12,2)	10,9 (6,7–17,4)	6,0 (2,9–12,2)
	Sim	43,9 (37,4–50,7)	55,6 (49,8–61,2)	53,1 (44,9–61,2)	53,6 (44,0–62,9)	47,1 (37,2–57,3)

IC95%: intervalo de confiança de 95%; -: menos de 25 observações.

**Figura 1:** Diferenças regionais na percepção materna negativa da saúde bucal das crianças, segundo quintis de riqueza, SB Brasil, 2023.

A relação entre o SII e a prevalência de percepção materna negativa da saúde bucal infantil, segundo subgrupos de raça/cor da pele, indica maior magnitude de desigualdade entre crianças brancas, apesar de esse grupo apresentar a menor prevalência do desfecho. Em contraste, crianças negras apresentaram a maior prevalência, porém com menor magnitude de desigualdade entre os quintis de riqueza (Figuras 2 e 3).

**Figura 2:** Diferenças regionais na percepção materna negativa da saúde bucal das crianças, SB Brasil, 2023. O eixo X apresenta a prevalência (%) de percepção materna negativa em cada região, com intervalos de confiança de 95% (IC 95%), e o eixo Y apresenta o Índice Absoluto de Desigualdade (*Slope Index of Inequality* – SII) segundo quintis de riqueza, também com IC 95%. Essa apresentação permite a visualização simultânea tanto da prevalência global quanto da magnitude da desigualdade entre as regiões do país.

**Figura 3:** Desigualdades na percepção materna negativa da saúde bucal das crianças segundo raça/cor da pele. O eixo X apresenta a prevalência (%) de percepção materna negativa em cada grupo racial/étnico, com intervalos de confiança de 95% (IC 95%), e o eixo Y apresenta o Índice Absoluto de Desigualdade (*Slope Index of Inequality* – SII) segundo quintis de riqueza, também com IC 95%. Essa apresentação permite a visualização simultânea da prevalência global e da magnitude das desigualdades entre os grupos de raça/cor da pele.

## DISCUSSÃO

Os resultados evidenciam disparidades socioeconômicas persistentes e marcantes na percepção materna da saúde bucal infantil no país. A percepção negativa mostrou-se mais frequente entre os grupos socialmente desfavorecidos. Ademais, a elevada prevalência de percepção materna negativa entre crianças negras, em média, em todos os quintis de riqueza, constitui um achado relevante. Esses padrões refletem desigualdades históricas nas condições de saúde bucal e reforçam a influência dos determinantes socioeconômicos na forma como as mães percebem e vivenciam a saúde bucal de seus filhos. Conforme descrito na literatura, esses grupos também apresentam maiores prevalências de cárie dentária e de cárie não tratada, em comparação com crianças cujas mães possuem níveis mais elevados de escolaridade^12^, pertencentes a quintis de riqueza mais altos^13,14^ e de outras raças/cores de pele^15^.

É fundamental reconhecer as limitações e os pontos fortes deste estudo para assegurar a interpretação adequada dos resultados. Em primeiro lugar, o reduzido número de observações de crianças indígenas e asiáticas limitou a realização de análises robustas segundo raça/cor da pele. Em segundo lugar, a variável de desfecho baseou-se em relatos maternos, e não na autopercepção das crianças, o que pode introduzir viés de informação, uma vez que tende a refletir mais as experiências e percepções sociais maternas do que as próprias experiências das crianças^16^. Esse método de mensuração pode ter levado à subestimação ou à superestimação do desfecho, a depender do grau de concordância entre a percepção materna e a experiência da criança. No entanto, considerando a faixa etária das crianças avaliadas, os relatos parentais constituem uma abordagem metodologicamente apropriada e amplamente empregada na literatura^6,7,17^. Por fim, algumas regiões do país apresentaram intervalos de confiança amplos, o que reduz a precisão das estimativas.

Outra limitação relevante refere-se à ausência de informações sobre a raça/cor da pele materna, o que pode ter restringido uma compreensão mais abrangente das desigualdades observadas. A origem racial/étnica materna influencia experiências sociais, interações com instituições e acesso aos serviços de saúde, podendo impactar práticas de cuidado, comportamentos de busca por atenção à saúde e percepções acerca da saúde da criança, repercutindo, em última instância, nos desfechos em saúde infantil.

Assim, a consideração exclusiva da raça/cor da pele da criança pode levar à subestimação do contexto social mais amplo que sustenta as desigualdades em saúde^18,19^.

O uso de uma medida subjetiva pode ser considerado uma limitação, uma vez que não permite inferências conclusivas acerca de piores condições de saúde bucal em crianças e apresenta menor precisão em comparação com variáveis clínicas. No entanto, essa abordagem pode ser empregada como indicador da saúde bucal infantil em situações nas quais a realização de exames clínicos não é viável, sendo mais econômica, prática e independente do uso de instrumentos clínicos^20,21^. Adicionalmente, nesta amostra, a prevalência de percepção materna negativa da saúde bucal infantil foi quatro vezes maior entre crianças com cárie dentária não tratada, em comparação àquelas sem cárie. Por outro lado, destacam-se como pontos fortes o uso de dados representativos em nível nacional, conferindo robustez metodológica ao estudo, bem como a adoção de processo de amostragem confiável, a utilização de examinadores padronizados e calibrados, a ampla cobertura populacional e a atualidade dos dados.

O presente estudo evidencia uma lacuna significativa entre os grupos de menor e maior renda, bem como entre aqueles com menor e maior escolaridade, na percepção materna negativa da saúde bucal infantil. Esse achado está em consonância com a literatura, a qual demonstra que indivíduos com menor renda e escolaridade enfrentam maiores barreiras de acesso a serviços odontológicos diante de condições como dor dentária ou cárie^22^. Tais barreiras incluem limitações financeiras relacionadas ao deslocamento até unidades de saúde, ao custeio do tratamento e ao próprio acesso ao atendimento, além de níveis mais baixos de alfabetização em saúde ^23^. Ademais, desfechos adversos, como a presença de cárie^15^, dor dentária^24^ e menor frequência de consultas odontológicas^25^, encontram-se associados à percepção materna negativa da saúde bucal infantil.

Considerando-se as diferentes regiões do país, a maior prevalência de percepção materna negativa da saúde bucal infantil foi observada entre crianças de mães negras e pardas. No entanto, identificaram-se importantes diferenças regionais, com distinções marcantes entre as regiões Norte, Nordeste e Centro-Oeste, em comparação às regiões Sul e Sudeste. Padrão semelhante foi observado em relação aos níveis de escolaridade materna. A maior prevalência de percepção materna negativa da saúde bucal infantil foi registrada na região Nordeste, enquanto a menor ocorreu na região Sul. De forma consistente, as maiores prevalências de cárie dentária não tratada e de experiência de cárie também foram observadas no Nordeste. Essa região caracteriza-se por apresentar menores níveis de renda no país^26^ e elevada vulnerabilidade socioeconômica, fatores que provavelmente contribuem para piores desfechos de saúde bucal entre as crianças e para uma percepção materna mais negativa. A persistente heterogeneidade socioeconômica no território nacional não apenas reforça as desigualdades no acesso aos serviços de saúde^27^, como também sugere que variações na implementação de programas relevantes de saúde bucal possam constituir fator adicional associado à pior percepção materna nessa região^28^.

A prevalência de percepção materna negativa evidencia disparidades marcantes segundo raça/cor da pele no Brasil. Crianças negras apresentaram maior prevalência em comparação às crianças brancas, embora a diferença tenha sido menor quando comparada à prevalência observada entre crianças pardas. Esse padrão consistente de percepção negativa evidencia desigualdades estruturais profundas e o racismo vivenciado por essas populações, os quais impactam negativamente diversas dimensões da saúde e da qualidade de vida^6,29^. Em muitos contextos, o preconceito manifesta-se também de forma institucional, no âmbito do próprio sistema de saúde, no qual profissionais podem reproduzir práticas discriminatórias e tratamento desigual^30–32^. Tal cenário agrava as barreiras de acesso e compromete a qualidade da atenção recebida por populações negras e pardas. Esse contexto reforça a importância da formulação e do fortalecimento de políticas públicas voltadas à promoção da equidade no acesso aos serviços de saúde bucal, bem como da implementação efetiva de políticas já existentes, como a Política Nacional de Saúde Integral da População Negra^33,34^.

Em conclusão, o presente estudo fornece evidências representativas em nível nacional sobre a persistência de desigualdades socioeconômicas e raciais na percepção materna da saúde bucal infantil no Brasil. Os achados indicam que a escolaridade materna, a renda familiar e a raça/cor da pele permanecem como determinantes centrais da forma como as mães avaliam o estado de saúde bucal de seus filhos, refletindo desigualdades socioeconômicas mais amplas que transcendem as condições clínicas. Esses resultados reforçam a importância da incorporação de abordagens orientadas para a equidade na vigilância em saúde bucal e no planejamento de políticas públicas, especialmente em regiões de maior vulnerabilidade socioeconômica. A ampliação do acesso à atenção preventiva, o fortalecimento da alfabetização em saúde bucal entre os cuidadores e a garantia de práticas culturalmente sensíveis e inclusivas no Sistema Único de Saúde (SUS) configuram estratégias essenciais para a promoção de desfechos mais justos e equitativos em saúde bucal para todas as crianças brasileiras.

## Material suplementar

Material suplementar 1
